# A unique binding mode of P1′ Leu-containing target sequences for *Streptococcus pyogenes* sortase A results in alternative cleavage†

**DOI:** 10.1039/d3cb00129f

**Published:** 2023-10-18

**Authors:** Brandon A. Vogel, Jadon M. Blount, Hanna M. Kodama, Noah J. Goodwin-Rice, Devin J. Andaluz, Sophie N. Jackson, John M. Antos, Jeanine F. Amacher

**Affiliations:** a Department of Chemistry, Western Washington University, 516 High St – MS9150 Bellingham WA 98225 USA antosj@wwu.edu amachej@wwu.edu +1-360-650-2826 +1-360-650-2271 +1-360-650-4397

## Abstract

Sortase enzymes are cysteine transpeptidases that attach environmental sensors, toxins, and other proteins to the cell surface in Gram-positive bacteria. The recognition motif for many sortases is the cell wall sorting signal (CWSS), LPXTG, where X = any amino acid. Recent work from ourselves and others has described recognition of additional amino acids at a number of positions in the CWSS, specifically at the Thr (or P1) and Gly (or P1′) positions. In addition, although standard cleavage occurs between these two residues (P1/P1′), we previously observed that the SrtA enzyme from *Streptococcus pneumoniae* will cleave after the P1′ position when its identity is a Leu or Phe. The stereochemical basis of this alternative cleavage is not known, although homologs, *e.g.*, SrtA from *Listeria monocytogenes* or *Staphylococcus aureus* do not show alternative cleavage to a significant extent. Here, we use protein biochemistry, structural biology, and computational biochemistry to predict an alternative binding mode that facilitates alternative cleavage. We use *Streptococcus pyogenes* SrtA (spySrtA) as our model enzyme, first confirming that it shows similar standard/alternative cleavage ratios for LPAT**L**, LPAT**F**, and LPAT**Y** sequences. Molecular dynamics simulations suggest that when P1′ is Leu, this amino acid binds in the canonical S1 pocket, pushing the P1 Thr towards solvent. The P4 Leu (L̲PATL) binds as it does in standard binding, resulting in a puckered binding conformation. We use P1 Glu-containing peptides to support our hypotheses, and present the complex structure of spySrtA-LPALA to confirm favorable accommodation of Leu in the S1 pocket. Overall, we structurally characterize an alternative binding mode for spySrtA and specific target sequences, expanding the potential protein engineering possibilities in sortase-mediated ligation applications.

## Abstract 2

Vogel *et al.* reveals the stereochemical basis of alternative substrate cleavage by *S. pyogenes* SrtA for ligands with a P1′ Leu residue. The substrate adopts puckered alternative binding, whereby cleavage occurs between the P1′ and P2′ positions.

## Graphical abstract

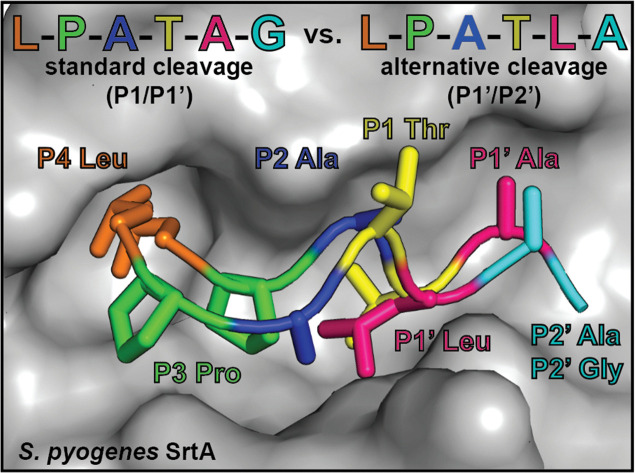

## Introduction

Bacterial sortases are cysteine transpeptidases located on Gram-positive bacteria, *e.g.*, *Staphylococcus aureus* and *Streptococcus pyogenes.* These enzymes catalyze a transacylation reaction that attaches proteins, including toxins and environmental sensors, to the peptidoglycan layer at the cell surface.^1–5^ The first sortase enzyme, Sortase A (SrtA) from *S. aureus*, was discovered in 1999.^4,6^ This enzyme is selective for the cell wall sorting signal (CWSS) sequence LPXTG, where X = any amino acid.^1,2,4,6^ Because SrtA is generally only specific for the positions of the CWSS, sortase-mediated ligation (SML) strategies have become powerful protein engineering tools for a variety of applications. Recently, these include development of SARS-CoV-2 vaccines and a diagnostic and therapeutic tool for amyloid-β aggregates in cerebral spinal fluid.^7,8^ SrtA enzymes are also potential novel antibiotic targets, which may be powerful aids against pathogens, *e.g.*, methicillin-resistant *S. aureus* (MRSA).^9–17^

**Fig. 1:**
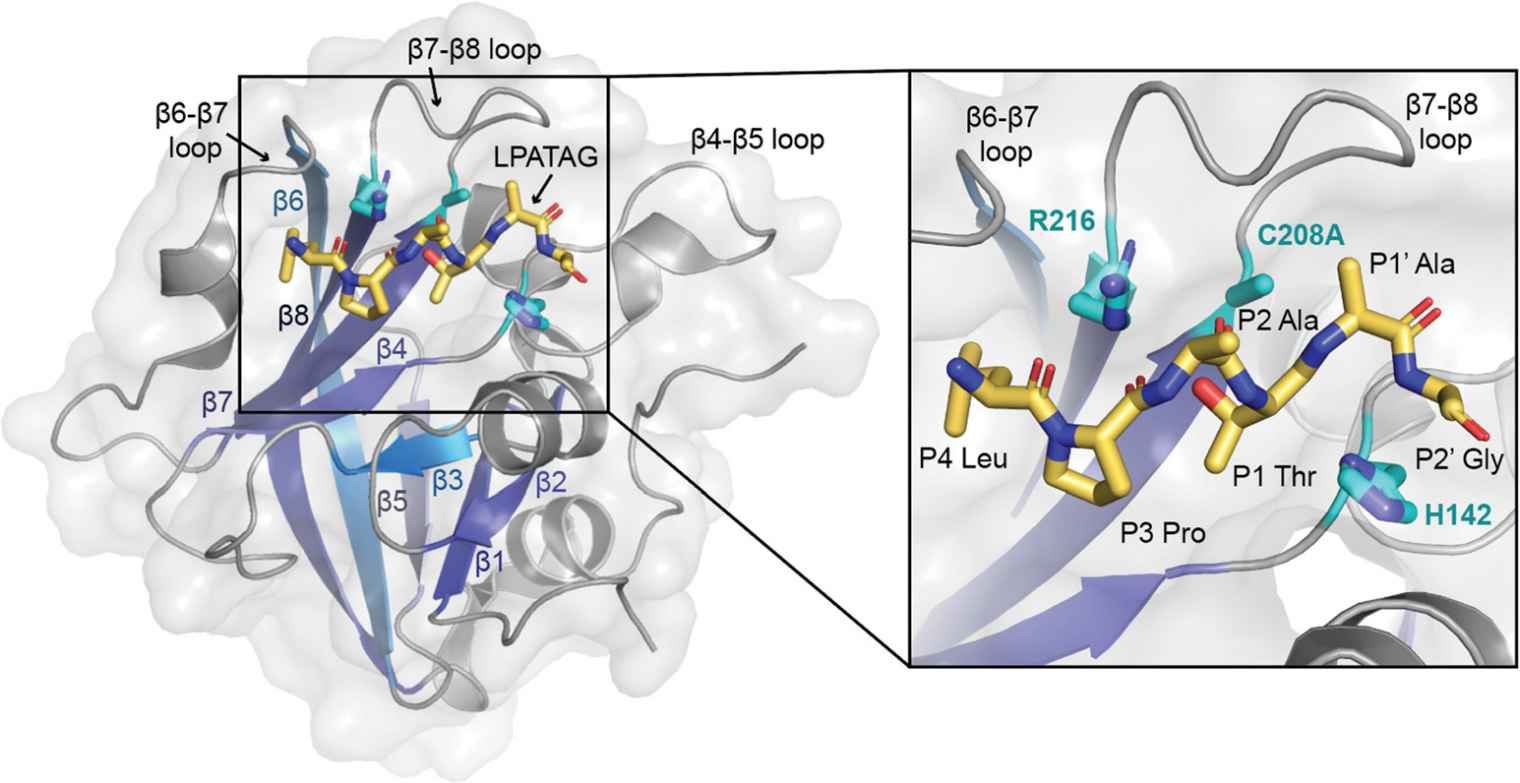
The overall sortase fold and target recognition by spySrtA. The C208A spySrtA protein is shown in cartoon, with a transparent gray surface (PDB 7S51).^5^ Conserved secondary structure elements that define the sortase fold are labeled and in different shades of blue. The LPATAG peptide is in yellow sticks and colored by heteroatom (N = blue, O = red). Peptide positions are labeled in the zoomed-in image. The catalytic residue side-chains are in cyan and colored by heteroatom. The three structurally-conserved loops near the active site are labeled.

SrtA enzymes are generally considered to be housekeeping sortase enzymes, in that they attach a wide variety of proteins responsible for performing many functions, and are present in most Gram-positive bacteria.^1^ These enzymes contain a highly conserved His-Cys-Arg triad that is required for activity in all wild-type SrtA enzymes characterized to date (Fig. 1).^1,2,4,6,18,19^ Upon recognition of the CWSS, the Cys thiol undergoes nucleophilic attack of the peptide bond between the P1 Thr and P1′ Gly residues (other residues are: P2 = X, P3 = P, P4 = L), forming an acyl–enzyme intermediate with the carbonyl C of the P1 Thr and cleaving the peptide.^1,2,20^ Resolution of this intermediate occurs when a second substrate, a Gly-containing nucleophile, attacks the acyl–enzyme intermediate and cleaves the C–S bond between the P1 Thr and catalytic Cys.^1,2,21^ The resulting ligation product consists of the LPAT of the initial substrate covalently linked to G of the second substrate through a native peptide bond.^1,2^ We recently determined a number of structures of *S. pyogenes* SrtA (spySrtA) bound to substrate peptides and a product mimic to elucidate details of the catalytic mechanism of SrtA.^22^ Interestingly, while *S. aureus* SrtA is absolutely specific for a P1′ Gly, spySrtA can recognize a number of amino acids at this position, as characterized previously by ourselves and others.^22–26^ This is not unique to spySrtA; indeed, these previous studies suggest varying levels of substrate promiscuity in P1 and P1′ for several sortase enzymes.^22–26^

As part of our previous sortase substrate profiling work, we reported an interesting phenomenon involving the site of substrate cleavage. Specifically, *Streptococcus pneumoniae* SrtA was able to catalyze an alternative cleavage reaction between the P1′ and P2′ residues, when P1′ = Leu or Phe.^22^ This effect was very dramatic for P1′ = Leu, with a standard/alternative cleavage ratio of 1 : 11.^22^ In contrast, for P1′ = Phe, the standard/alternative cleavage ratio was 1 : 1.^22^ In another enzyme, *Listeria monocytogenes* SrtA (lmSrtA), the standard/alternative cleavage ratio for P1′ = Phe was 25 : 1, suggesting little-to-no alternative cleavage despite lmSrtA recognition of the LPAT**F** sequence.^22^ Given that all SrtA enzymes share the conserved sortase fold, consisting of an 8-stranded antiparallel β-barrel (Fig. 1), these findings were particularly striking and suggested a novel mode of substrate binding, or possibly even an alternate catalytic mechanism.^5^ However, a detailed stereochemical understanding of this alternative cleavage process was not elucidated at that time.

Here, we use protein biochemistry, structural biology, and computational biochemistry to understand alternative cleavage in spySrtA, and likely other *Streptococcus* SrtA enzymes. We use activity assays with a number of wild-type and mutant spySrtA and lmSrtA enzymes to show that the catalytic mechanism is likely largely intact. We use molecular dynamics simulations to build upon our previous observation that these enzymes are able to bind substrates with a P1 or P1′ Leu, and in cases of alternative cleavage, the P1′ Leu docks in the canonical S1 site.^22^ In this alternative binding mode, the P4 Leu binds in its traditional site; therefore, we predict the peptide is in a puckered conformation. In this conformation the catalytic mechanism may proceed as in the standard cleavage scenario, however the end result is cleavage between the P1′ and P2′ residues. Although we predict the strain introduced by the puckered peptide results in a spectrum of cleavage activity for different P1 amino acids, this creates an alternative motif for spySrtA of LPXXLG, which introduces new potential for future SML applications.

## Materials and methods

### Expression and purification of sortase proteins

Wild-type (WT) spySrtA (residues 81–249, corresponding to PDB 3FN5), WT lmSrtA (corresponding to residues 71–222, UniProt ID SRTA_LISMO, PDB 5HU4), H143A spySrtA, I211P spySrtA, C208A spySrtA, and the β7–β8 loop chimeric proteins, spySrtA_monocytogenes_ and lmSrtA_pyogenes_ sequences were expressed using the pET28a(+) plasmid (Genscript) in *Escherichia coli* BL21 (DE3) cells, as described previously.^5,22,23,25,27^ All plasmids contained a 6xHis tag followed by TEV cleavage site (sequence: ENLYFQS). The purification protocols were also those used previously.^5,23,25,28^ Briefly, following induction by IPTG and overexpression, the cells were harvested in lysis buffer [0.05 M Tris pH 7.5, 0.15 M NaCl, 0.5 mM ethylenediaminetetraacetic acid (EDTA)], lysed *via* sonication, and clarified using centrifugation, followed by filtration of the supernatant. Initial purification was conducted *via* immobilized metal affinity chromatography (IMAC) using a 5 mL HisTrap HP column (Cytiva), with wash [0.05 M tris pH 7.5, 0.15 M NaCl, 0.02 M imidazole, 0.001 M TCEP] and elution [wash buffer with 0.3 M imidazole] buffers.

Proteins used in activity assays retained their 6xHis-TEV sequences. Following IMAC, the elution was concentrated using an Amicon Ultra-15 Centrifugal Filter Unit (10 000 NWML) and size exclusion chromatography (SEC) was conducted using a HiLoad 16/600 Superdex 75 column (Cytiva) in SEC running buffer [0.05 M Tris pH 7.5, 0.15 M NaCl, 0.001 M TCEP]. For crystallography, C208A spySrtA was subjected to TEV protease cleavage overnight and the flow-through was collected from a second 5 mL HisTrap HP column [wash buffer identical to that described above], prior to SEC. Protein not immediately used was flash-frozen in SEC running buffer and stored at −80 °C.

The purity, monomeric state, and identity of purified enzymes were confirmed by SDS-PAGE, analytical SEC, and LC-ESI-MS (Table S1, ESI†). Protein concentrations were determined using theoretical extinction coefficients calculated using ExPASy ProtParam.^29^ The following extinction coefficients were used to determine protein concentrations following absorbance measurements at *λ* = 280 nm: all spySrtA variants = 11 920 M^−1^ cm^−1^, all lmSrtA variants = 10 430 M^−1^ cm^−1^.

### Peptide synthesis

Peptide substrates used in crystallization and/or enzyme assays contained the general structure: Abz-LPAX̲X̲GK(Dnp), where Abz = 2-aminobenzoyl, Dnp = 2,4-dinitrophenyl, NH_2_ = C-terminal primary amide. Peptides were synthesized and purified as previously described.^5,25^

### Crystallization and structure determination of C208A spySrtA-LPALA

Crystallization of C208A spySrtA with Abz-LPALAGK(Dnp)-NH_2_ (abbreviated to LPALA) followed a similar protocol to that used for spySrtA-LPATA (PDB 7S51), spySrtA-LPATS (7S4O), and spySrtA-LPAT-LII (7T8Y, 7T8Z).^5^ Briefly, approximately 10 mg mL^−1^ (or 0.5 mM) C208A spySrtA was incubated with 1 mM peptide (2.5% (v/v) DMSO) for approximately 1 h prior to crystallization by hanging drop vapor diffusion. Crystal drops included 2 μL of the protein–peptide solution plus 2 μL of the crystallization well solution. Crystals appeared after 2–3 days and grew for approximately 1 week before harvesting. The crystal used for data collection grew in 0.25 M sodium acetate, 30% (w/v) PEG 8000, 0.1 mM Tris pH 6. The cryoprotectant solution used was 0.15 M sodium acetate, 10% (w/v) PEG 8000, 40% (w/v) PEG 400, 0.1 M tris pH 6. The crystals were flash cooled by plunging into liquid nitrogen.

Diffraction data were collected at the Advanced Light Source at Lawrence Berkeley National Laboratory on beamline 5.0.1, at *λ* = 0.97741 over 360°, with Δ*Φ* = 0.25° frames and an exposure time of 0.5 s per frame. Data were processed using the XDS package (Max Planck Institute for Medical Research), scaling using Aimless (CCP4i), and molecular replacement using Phenix, with PDB 7S51 (spySrtA-LPATA) as the search model.^30–32^ Refinement was performed using Phenix, and manual refinement using Coot.^33^ Model geometry was assessed using the Molprobity server and the PDB validation server.^34^ Final data collection and refinement statistics are in Table 2. The spySrtA-LPALA structure is deposited in the Protein Data Bank with PDB accession code 8T8G.

### Activity assays using HPLC/LC-MS

Enzyme activity assays were performed with 200 μM peptide substrate and 25 μM sortase enzyme. All reactions also contained 10% (v/v) sortase reaction buffer (500 mM Tris pH 7.5, 1500 mM NaCl), 10 mM hydroxylamine, and 10 mM CaCl_2_. Activity assays were run with CaCl_2_ in the reaction buffer to maintain reaction conditions consistent with our previous study that initially identified alternative cleavage; however, this likely has no effect on spySrtA activity.^22,23,25,35^ Reactions also contained 5% (v/v) residual DMSO from peptide stock solutions. Reactions were run at room temperature and monitored using an Agilent AdvanceBio 6545XT Q-TOF mass spectrometer interfaced with an Agilent 1290 UHPLC. Separations upstream of the mass spectrometer were achieved with a Phenomenex Kinetex C18 column (2.6 μm, 100 Å, 100 × 2.1 mm). Samples were separated using a H_2_O/MeCN (0.1% v/v formic acid) mobile phase with a linear gradient of 10–90% MeCN. Data was analyzed using the Chemstation Masshunter software suite.

The transacylation reaction between Abz-LPAELGK(Dnp) and glycinamide was performed with 50 μM peptide substrate, 20 μM wild-type spySrtA, and 5 mM glycinamide. The reaction also contained 10% (v/v) sortase reaction buffer (500 mM Tris pH 7.5, 1500 mM NaCl). The reaction was incubated at room temperature for 24 h and analyzed by HPLC/LC-MS as described above.

### Molecular dynamics simulations

All MD simulations were performed as described previously.^5^ Briefly, GROMACS 2020.4 (GROMACS development teams at the KTH Royal Institute of Technology and Uppsala University) with the AMBER99SB*-ILDN force fields was used and simulations were run for 1000 ns.^36–40^ The starting protein structures were solvated with TIP3P water molecules in a cubic box with periodic boundary conditions, and using a neutralizing ionic concentration of 150 mM (Table S2, ESI†). All peptides in the starting models were “capped” by acetylation on the N-terminus and amidation (*N*-methyl amide) on the C-terminus to neutralize charges. The N-terminus of spySrtA was also acetylated; however, because the C-terminus of the protein was included in all tested proteins, we kept the charge on the terminal residue in our simulations. The system was first equilibrated in an *NVT* ensemble for 100 ps, then in an *NPT* ensemble for 5000 ps. The starting models used for each simulation were derived from spySrtA-LPATA (7S51), and peptide residues were mutated (and/or shifted) using Coot.

### Programs used for protein analyses

BLASTP was used for pairwise sequence alignments.^41^ We used PyMOL to render all figures.

## Results and discussion

### Activity assays with wild-type and mutant SrtA proteins

We previously reported that *S. pneumoniae* SrtA and lmSrtA are capable of recognizing large, hydrophobic amino acids (Phe, Leu, Tyr) at the P1′ position of the LPXTG̲ cell wall sorting signal motif.^22^ These previous reactions were conducted at room temperature for 24–26 h with 25 μM sortase and 200 μM substrate, amongst other buffer components. The general form of all peptides in these experiments was Abz-LPAT**X**GK(Dnp), but will be abbreviated to the pentapeptide motif (here, LPAT**X**) moving forward for simplicity. As we previously reported, for *S. pneumoniae* SrtA, substrate conversions for P1′ Phe, Leu, and Tyr, respectively were: 42%, 38%, and 24%.^22^ The related conversion values for lmSrtA were: 31%, 2%, 45% for Phe, Leu, and Tyr, respectively.^22^ In these experiments lmSrtA showed only trace levels of alternative cleavage, defined as occurring between the P1′ and P2′ residues, or LPAT**X**/G in our peptides, with standard/alternative cleavage ratios of 25 : 1 for LPAT**F** and 30 : 1 for LPAT**Y**. Interestingly, *S. pneumoniae* SrtA revealed a different result, with standard/alternative cleavage ratios of 1 : 1, 1 : 11, and 3 : 1 for LPAT**F**, LPAT**L**, and LPAT**Y**, respectively.^22^ We were therefore interested in determining the mechanism of the observed alternative cleavage, a characteristic that appeared to be specific for *S. pneumoniae* SrtA in these studies.

**Fig. 2:**
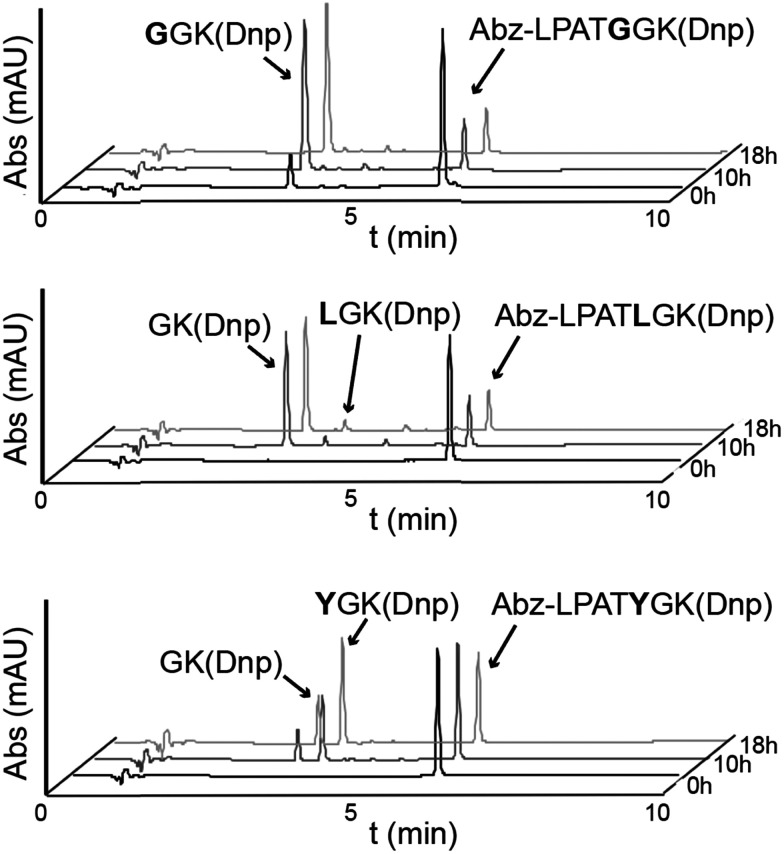
Activity assays reveal alternative cleavage for spySrtA and a target sequence containing P1′ Leu or Tyr. RP-HPLC monitoring (360 nm) was used to detect Dnp-containing reaction products and to calculate the extent of substrate cleavage. The reactions shown were run for 18 h, and the identity of reaction products was confirmed by mass spectrometry. Each graph is a representative example of spySrtA, with either the peptide sequences Abz-LPATGGK(Dnp) (top), Abz-LPATLGK(Dnp) (middle), or Abz-LPATYGK(Dnp) (bottom), where K(Dnp) indicates a Lys residue with a 2,4-dinitrophenyl (Dnp) chromophore attached to the amine side chain. The summary of triplicate data is in Table 1. Alternative cleavage is indicated by the “GK(Dnp)” peaks, whereas standard cleavage produces GGK(Dnp), LGK(Dnp), and YGK(Dnp), respectively. The ratio of standard to alternative cleavage is 1 : 13 for LPATL and 2 : 1 for LPATY, respectively. No alternative cleavage was observed for LPATG.

We first set out to replicate the alternative cleavage process using the SrtA enzymes from *S. pyogenes* and lmSrtA. Our recent work revealed that spySrtA is much more active than *S. pneumoniae* SrtA, but shares many of the same selectivity characteristics.^23^ We expressed and purified the soluble domains of recombinant spySrtA (residues 81–249) and lmSrtA (residues 71–222) as previously described, and as in the Materials and Methods (Table S1, ESI†).^5,23^ Reactions of SrtA with the peptides, LPAT**G**, LPAT**L**, and LPAT**Y**, were run as previously described, and as in the Materials and methods, including the use of excess hydroxylamine to serve as the reaction nucleophile.^5,6,22,25^ The extent of substrate cleavage, which is the first step in the overall transacylation reaction, was determined by reversed-phase high-performance liquid chromatography (RP-HPLC) (Fig. 2 and Table 1(A)). Relative levels of reaction components containing the 2,4-dinitrophenyl (Dnp) chromophore were monitored, allowing for estimation of overall substrate cleavage, as well as the ratio of standard (P1/P1′) *versus* alternative (P1′/P2′) cleavage for each substrate and enzyme pair (Table 1(B)). In all cases, the identities of relevant Dnp-containing peptide species were confirmed by LC-MS (Table S1, ESI†).

Relative activities of sortase enzymes based on percentage of substrate cleaved in an 18 h assay. (A) Average substrate cleavage (*N* = 3) and standard deviation shown. Where appropriate, total percent cleavage is separated into amounts corresponding to standard (P1/P1′) *versus* alternate (P1′/P2′) cleavage products. (B) Calculated ratio of standard (P1/P1′) to alternative (P1′/P2′) cleavage. “—” indicates that cleavage products were not observed

**Table:** 

(A)						
	% of input substrate converted to product					
	LPAT**G**		LPAT**L**		LPAT**Y**	
	P1/P1′	P1′/P2′	P1/P1′	P1′/P2′	P1/P1′	P1′/P2′
WT spySrtA	80.7 ± 0.6	—	5.6 ± 0.3	73.6 ± 2.3	42.7 ± 1.2	18.2 ± 0.9
H143A spySrtA	77.8 ± 0.4	—	4.3 ± 0.7	41.2 ± 7.1	10.8 ± 0.4	7.3 ± 0.3
I211P spySrtA	77.9 ± 2.1	—	7.7 ± 0.4	62.3 ± 5.7	34.7 ± 0.9	18.2 ± 0.7
spySrtA_mono_	78.4 ± 0.5	—	26.7 ± 2.3	23.6 ± 1.7	67.3 ± 0.7	3.6 ± 0.5

WT lmSrtA	75.4 ± 2.0	—	5.4 ± 0.2	6.8 ± 1.6	40.8 ± 1.2	1.3 ± 0.3
lmSrtA_pyogenes_	21.9 ± 0.1	—	—	—	—	—

**Table:** 

(B)			
	Ratio of standard/alternative cleavage		
	LPAT**G**	LPAT**L**	LPAT**Y**
WT spySrtA	—	1 : 13	2 : 1
H143A spySrtA	—	1 : 10	1.5 : 1
I211P spySrtA	—	1 : 8	2 : 1
spySrtA_mono_	—	1 : 1	19 : 1

WT lmSrtA	—	1 : 1	31 : 1
lmSrtA_pyogenes_	—	—	—

Focusing on the 18 h reaction endpoint, our results largely recapitulated previous findings, with no alternative cleavage observed for the LPAT**G** peptide for either WT enzyme, and standard/alternative cleavage ratios of 1 : 13 and 2 : 1 for spySrtA + LPAT**L** and LPAT**Y**, respectively, and 31 : 1 for lmSrtA + LPAT**Y** (Table 1). We did see a standard/alternative cleavage ratio of 1 : 1 for lmSrtA + LPAT**L**; however, it should be noted that the overall percentage of substrate consumed in this experiment was low (5.4 ± 0.2% for P1/P1′ standard cleavage, and 6.8 ± 1.6% for P1′/P2′ alternative cleavage) (Table 1).

We next wanted to assess if we could modulate these cleavage ratios with mutation. All expression and purification of variant proteins followed similar protocols as used previously, and as described in the Materials and methods (Table S1, ESI†).^23,25^ We tested two mutations in spySrtA: H143A, the histidine residue immediately following the catalytic His in the β4–β5 loop, as well as I211P, which mimics a position in the lmSrtA β7–β8 loop and likely disrupts a hydrophobic interaction between the β4–β5 and β7–β8 loops, as we previously characterized (Table 1).^5,23,25^ Finally, we also tested two β7–β8 loop chimera proteins, where we substituted the residues of lmSrtA into the β7–β8 loop of spySrtA (spySrtA_monocytogenes_) and *vice versa* (lmSrtA_pyogenes_) (Table 1). These chimeric variants were also based on our previous work that revealed activity and selectivity determinants in the β7–β8 loops of SrtA enzymes.^23,25^

We expressed, purified, and tested all variants as with the wild-type proteins. Beginning with the spySrtA point mutants (H143A and I211P), we found that these enzymes gave similar levels of substrate cleavage at 18 h as compared to wild-type spySrtA when assayed against the LPAT**G** sequence (Table 1(A)). Only P1/P1′ standard cleavage was observed in these reactions as well. When tested with LPAT**L** or LPAT**Y**, there were only modest changes in the standard/alternative cleavage ratios (Table 1(B)), however the total amount of substrate consumed was significantly lower for H143A spySrtA *versus* wild-type spySrtA or the I211P mutant. Therefore, while H143 appeared to play a significant role in the overall activity of spySrtA toward noncanonical substrates, both H143 and I211 were less critical for standard/alternate cleavage selectivity.

In contrast to the point mutations, results with the spySrtA_monocytogenes_ loop chimera were more distinct. While the overall amount of LPAT**G** consumed (78.4%) was comparable to wild-type spySrtA, the standard/alternative cleavage ratio for LPAT**L** and spySrtA_monocytogenes_ was 1 : 1 (26.7% for P1/P1′ and 23.6% for P1′/P2′) and 19 : 1 (67.3% for P1/P1′ and 3.6% for P1′/P2′) for LPAT**Y** (Table 1). This represented a significant drop in the observed levels of alternate *versus* standard cleavage as compared to wild-type spySrtA. Moreover, the observed ratios of standard/alternate cleavage for spySrtA_monocytogenes_ were more similar to wild-type lmSrtA, suggesting a critical role for the entire β7–β8 loop sequence in determining cleavage site selectivity. For the reverse loop chimera, lmSrtA_pyogenes_, activity was reduced by over 3-fold for LPAT**G** as compared to lmSrtA, and we did not observe any activity for either LPAT**L** nor LPAT**Y**. Taken together, these results provided evidence that residues in the β4–β5 and β7–β8 loops likely play a role in alternative cleavage by spySrtA, however we consider it unlikely that it is due to a fundamental change in the catalytic mechanism of the wild-type protein. Rather, we hypothesized that P1′/P2′ cleavage may be mediated by an alternative binding mode of the peptide.

### Molecular dynamics simulations of a shifted LPATLG peptide reveals alternate binding mode

In order to test the prediction that noncanonical substrate sequences may be positioned differently in the peptide-binding pocket, thus facilitating alternative cleavage, we turned to computational biochemistry and molecular dynamics simulations. Given that the highest levels of alternate cleavage were observed with LPAT**L**, we elected to focus on this motif. This was also based on our previous observation that 5 of 8 SrtA enzymes tested, including *Streptococcus suis*, *S. pneumoniae*, *L. monocytogenes*, *Enterococcus faecalis*, and *Lactobacillus lactis* SrtA, can recognize a P1 Leu as well or better than a P1 Thr.^22^ All molecular dynamics simulations were run for 1000 ns using similar methods to those previously described, and as in the Materials and methods (Table S2, ESI†).^5^ Using our spySrtA-LPAT**A** structure (PDB 7S51) as a template, we *in silico* shifted the peptide such that the P1′ position (mutated to Leu) sat in the S1 pocket, creating spySrtA-LPAT**L**A_shifted (Fig. S1A, ESI†). In parallel, we ran a simulation of spySrtA-LPA**L**A, again using PDB 7S51 as a template, and by *in silico* mutating the P1 residue (Fig. S1B, ESI†).

**Fig. 3:**
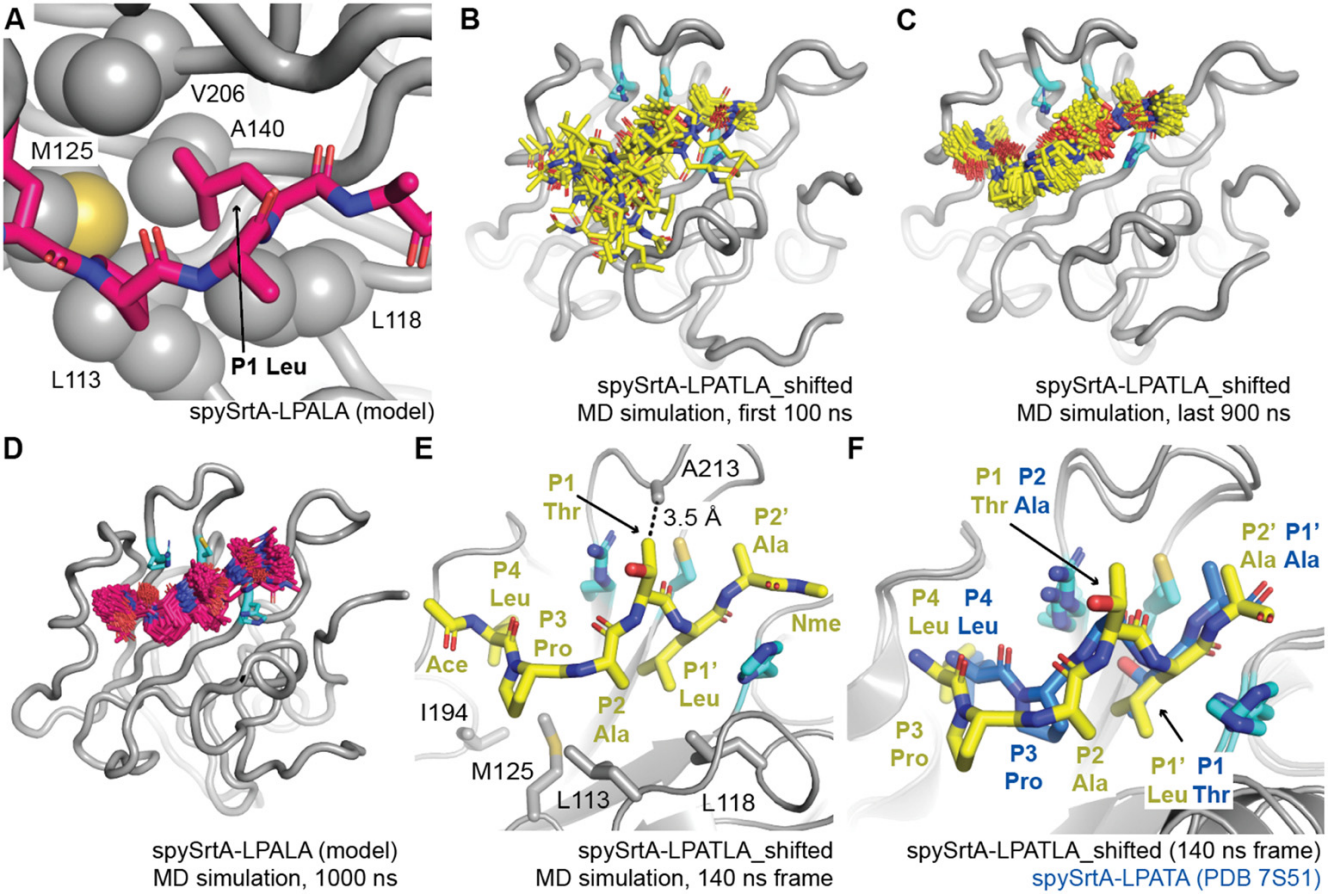
Molecular dynamics simulations reveal a unique binding mode that facilitates alternative cleavage. For all, the spySrtA protein is in gray cartoon, with relevant side chain atoms shown as sticks and labeled, with the exception of (A) where side chain sticks are shown as spheres. The catalytic residues are in cyan sticks and colored by heteroatom (N = blue, O = red, S = yellow). (A) The P1 Leu binds a hydrophobic pocket (the S1 site) in spySrtA. The LPALA peptide model, *in silico* mutated from spySrtA-LPATA (PDB 7S51) is in hot pink sticks and colored by heteroatom. (B–C) Frames, at 5 ns time intervals, for the 1000 ns MD simulation of spySrtA-LPATLA (*in silico* shifted so that the P1′ Leu is in the S1 pocket) are shown for the first 100 ns (B) and final 900 ns (C). The peptide is in yellow sticks and colored by heteroatom. (D) Frames, at 5 ns time intervals, for the 1000 ns MD simulation of spySrtA-LPALA are shown, colored as in (A). (E) and (F) Specific identified interactions in the spySrtA-LPATLA_shifted simulation are highlighted as labeled and described in the main text (E), and compared to the experimentally-determined spySrtA-LPATA structure (F). Here, Ace and Nme indicate the acetyl and *N*-methyl amide peptide caps present for all MD simulations.

Our simulations revealed that, as expected, a Leu is stable in the S1 binding pocket. Overall, the enzyme is very stable as well, and there were minimal fluctuations in the backbone of the spySrtA protein in both simulations (Fig. S2, ESI†). We predict that Leu stabilization in the S1 site is due to hydrophobic contacts with L113, L118, M125, A140, and V206 (Fig. 3(A)). Interestingly, although the P4 Leu (L̲PATLA) did not make stable contacts with spySrtA at the beginning of our spySrtA-LPAT**L**A_shifted simulation, based on our modeling, this residue finds its canonical S4 binding pocket within 100 ns of simulation time (Fig. 3(B)). The P4 Leu is then stable for the remainder of the simulation (Fig. 3(C)). This result is consistent with the spySrtA-LPAL̲A simulation, which revealed minimal binding fluctuations in the peptide throughout the simulation (Fig. 3(D)).

Of particular interest to us was the overall binding conformation of the peptide in the spySrtA-LPAT**L**A_shifted simulation once P4 Leu binding stabilized. Here, we observed that the peptide puckers out in order to accommodate the additional residue between the S4 and S1 binding sites. Specifically, the P2 Ala is shifted and binds in the canonical S3 Pro-binding site, likely stabilized by hydrophobic interactions with L113 and L118 (Fig. 3(E)). The P3 Pro is likely also still interacting with L113, and also being stabilized by hydrophobic interactions with M125 and I194. The P2′ Ala then binds in the S1′ Ala-binding site of PDB 7S51 (Fig. 3(F)). Perhaps, most intriguingly, the P1 Thr remained solvent-exposed for the entire simulation (Fig. 3(B) and (C)). This suggests that in this binding mode, this position may potentially be any amino acid. Notably, there is a potential van der Waals interaction between the side chain of A213 and Cγ of the puckered P1 Thr (Fig. 3(E)). Therefore, we were curious if we could modify the peptide sequence at the P1 residue and still observe alternative cleavage.

### Activity assays with alternate P1 amino acids

**Fig. 4:**
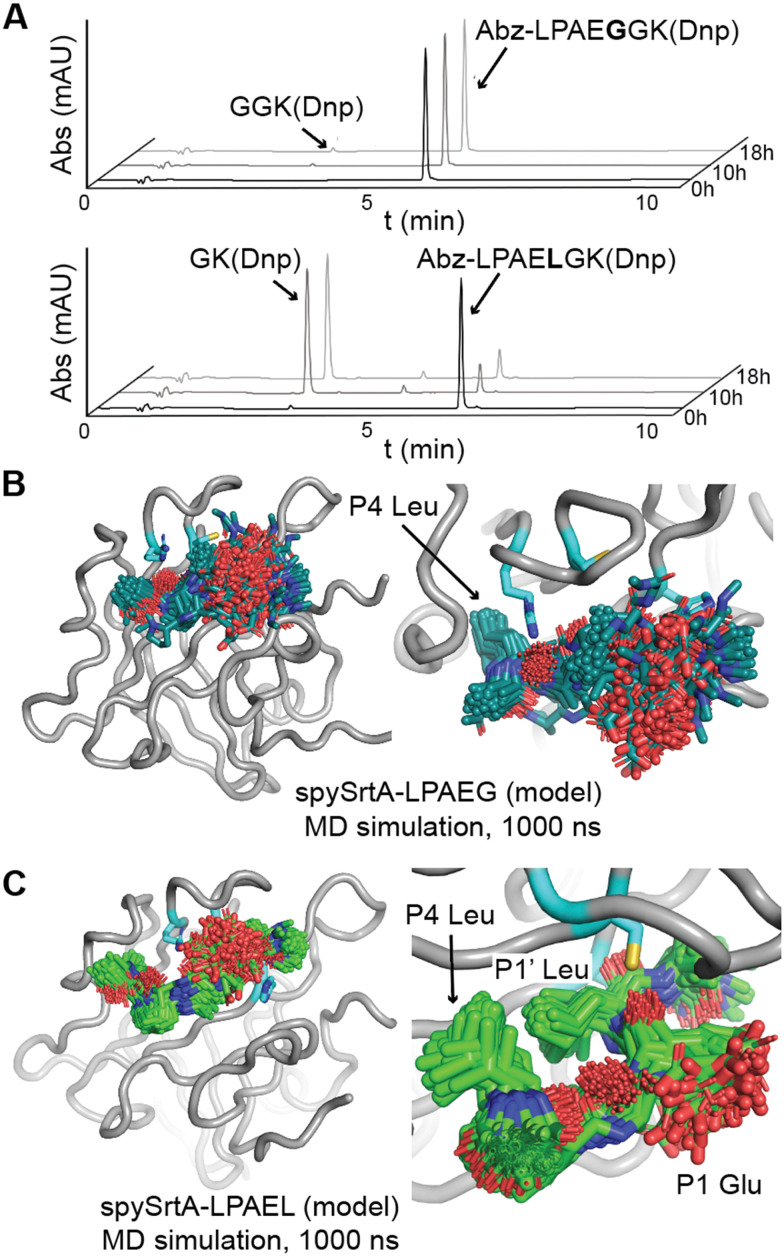
Accommodation of a P1 Glu in the alternative cleavage binding mode of spySrtA. (A) RP-HPLC monitoring (360 nm) was used to detect Dnp-containing reaction products and to calculate the extent of substrate cleavage. The reactions shown were run for 18 h, and the identity of reaction products was confirmed by mass spectrometry. Each graph is a representative example of spySrtA, with either the peptide sequences Abz-LPAEGGK(Dnp) (top) or Abz-LPAELGK(Dnp) (bottom), where K(Dnp) indicates a Lys residue with a 2,4-dinitrophenyl (Dnp) chromophore attached to the amine side chain. (B) and (C) The spySrtA protein is shown as a gray cartoon, with the catalytic residues in cyan sticks and colored by heteroatom (N = blue, O = red, S = yellow). The LPAEG peptide is in teal sticks (B) and LPAEL peptide in green sticks (C), both colored by heteroatom. Frames of the simulation at 5 ns intervals are shown for both simulations, and figures to the right indicate zoomed-in versions highlighting the P4 Leu stability (B) and P4 Leu/P1′ Leu stability (C) throughout the simulations.

Based on our observations in our molecular dynamics simulations, we wanted to test the hypothesis that an alternative binding mode, where the P1′ Leu of LPAT**L** sits in the S1 pocket, results in P1′/P2′ alternative cleavage for this sequence. We decided to test activity in a sequence with a P1 amino acid that is not recognized, reasoning that if spySrtA can bind the peptide in this alternative binding mode, the P1 position becomes nonselective, as it is solvent-exposed. Therefore, we synthesized and tested the peptides: Abz-LPAE̲**G**GK(Dnp) (LPAE̲**G**) and Abz-LPAE̲**L**GK(Dnp) (LPAE̲**L**), with a P1 Glu. The former peptide, LPAE̲**G**, showed no activity with any of the 8 sortase enzymes previously tested.^22^ An LPRE̲G sequence has also been shown to be unreactive with wild-type spySrtA, suggesting that Glu is generally not tolerated in the P1 position.^24^ We tested these two peptides with wild-type spySrtA and indeed, LPAE̲**G** exhibited very low reactivity, with only 2.9 ± 0.9% of the initial substrate being cleaved over 18 h in a triplicate experiment (Fig. 4(A)). In this case, LC-MS suggested that this was predominantly due to standard cleavage (P1/P1′) (Table S1, ESI†). In contrast, significant activity was observed with LPAE̲**L** (85.4 ± 0.7% cleavage, Fig. 4(A)). As anticipated, the LPAE̲**L** sequence gave rise to exclusive alternate cleavage (P1′/P2′), with no evidence of standard cleavage products by LC-MS (Table S1, ESI†).

Along with monitoring substrate cleavage, we also evaluated reactions between wild-type spySrtA and LPAE̲**L** for the formation of transacylation products involving the added hydroxylamine nucleophile. Consistent with the observation of exclusively alternate cleavage, HPLC and LC-MS only suggested transacylation products arising from initial substrate cleavage at the P1′/P2′ position (Fig. S3A, ESI†). Substrate hydrolysis was detected as well, however transacylation was favored by a ratio of 40 : 1 as estimated by RP-HPLC. For comparison, we conducted a similar analysis for reactions between wild-type spySrtA and LPAT̲**L**, which as described above also generated significant levels of alternate cleavage (Table 1(B)). Transacylation products arising from alternate cleavage (P1′/P2′) were indeed produced from the LPAT̲**L** substrate, however they were mixed with standard transacylation products derived from initial cleavage at the P1/P1′ position (Fig. S3B, ESI†). A related set of hydrolysis products derived from LPAT**L** was also found, corresponding to a ratio of 28 : 1 for the combined level of transacylation *versus* hydrolysis. Given that the LPAE̲**L** substrate resulted in higher selectivity for the alternate *versus* standard cleavage pathway, we also attempted a transacylation reaction in which hydroxylamine was replaced by the amine nucleophile glycinamide. Selective transacylation was once again observed, along with ∼4% competing hydrolysis, confirming that a simple amino acid derivative was compatible with transacylation at the alternate (P1′/P2′) site of the LPAE̲**L** substrate (Fig. S3C, ESI†). Interestingly, this reaction also showed trace levels of P1/P1′ cleavage products by mass spectrometry, but these components were otherwise undetectable in the HPLC chromatograms (Fig. S3C, ESI†).

To gain more insight into substrates with P1 Glu, we also ran 1000 ns molecular dynamics simulations of spySrtA-LPAE̲**G** and spySrtA-LPAE̲**L**A, again using spySrtA-LPAT**A** (PDB 7S51) as a template and *in silico* mutating the P1 and P1′ positions (Fig. S1C and D, ESI†). Again, the spySrtA protein was very stable in both simulations (Fig. S2, ESI†). In the spySrtA-LPAE̲**G** simulation, while the P4 Leu and P3 Pro residues were stable, the rest of the peptide was very flexible and there was no discernible binding, reflective of the biochemical assay results (Fig. 4(B)). Therefore, even if the peptide is able to bind spySrtA, it is likely that the P1/P1′ peptide bond will not be positioned properly for nucleophilic attack by the catalytic Cys. In contrast, the P4 and P1′ Leu residues anchored the peptide in the spySrtA-LPAE̲**L**A simulation, and the peptide bound in the alternative binding mode, with the P1′ Leu in the S1 pocket, throughout the entire 1000 ns simulation (Fig. 4(C)). Taken together, these results strongly support the alternative binding mode described here.

### Crystal structure of C208A spySrtA bound to LPALA peptide

**Fig. 5:**
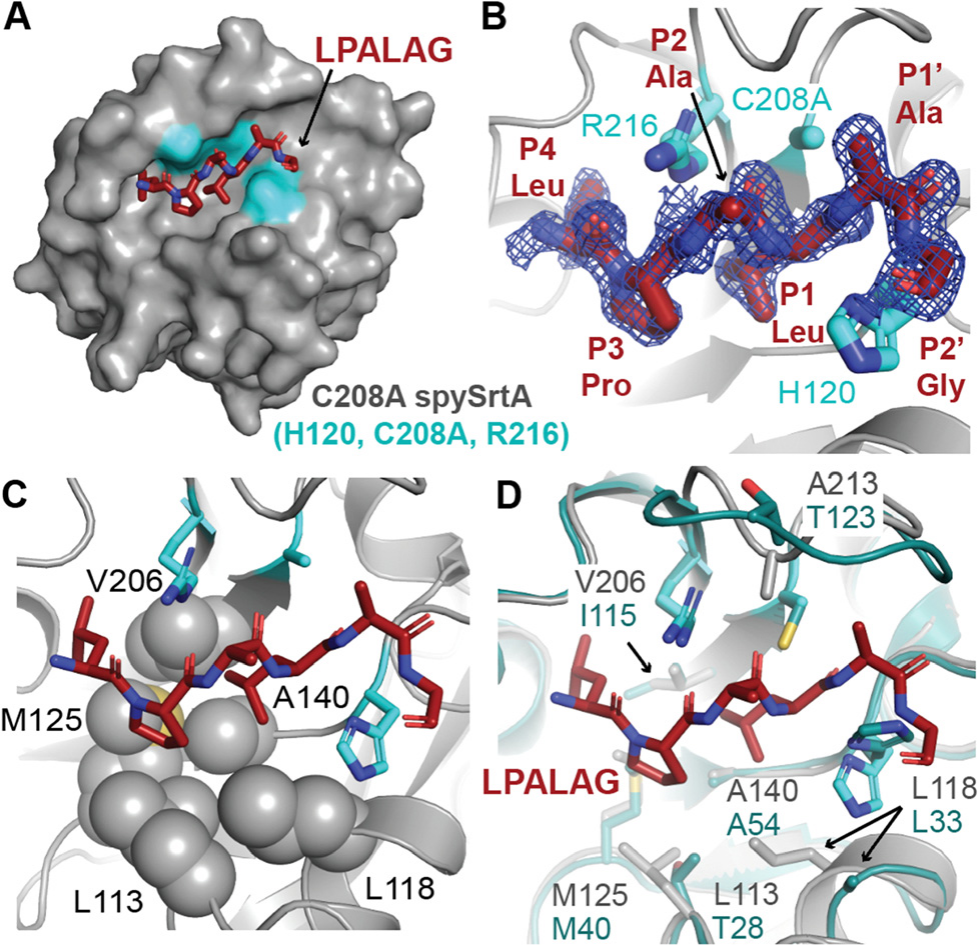
Crystal structure of C208A spySrtA-LPALA. (A) The surface representation of C208A spySrtA is shown in gray with the catalytic residues in cyan. The LPALAG peptide is in dark red sticks, colored by heteroatom (N = blue, O = red, S = yellow). (B) The 2*F*_o_ − *F*_c_ electron density map at 1*σ* reveals clear peptide density for all residues shown. The Abz and GK(Dnp) atoms of the peptide were not resolved. The peptide is colored as in (A) and catalytic residue side chains shown as sticks and colored by heteroatom. Residues are labeled. (C) The P1 Leu occupies the predicted S1 hydrophobic binding pocket predicted in Fig. 3(A), with relevant spySrtA side chains shown as spheres, colored by heteroatom. All other rendering is as in (B). (D) Comparison of spySrtA-LPALA with lmSrtA (PDB 5HU4).^27^ SpySrtA-LPALA is rendered and colored as in (B) and (C). LmSrtA is in teal, with relevant residue side chains shown as sticks, labeled, and colored by heteroatom. The main chain atoms of lmSrtA and spySrtA align with RMSD = 0.494 Å, over 397 atoms.

Finally, we wanted to experimentally determine a structure that included P1 Leu binding to spySrtA to support our observations of Leu binding in the S1 site. We crystallized and solved the structure of C208A spySrtA bound to Abz-LPAL̲AGK(Dnp). The C208A spySrtA protein was expressed and purified as previously described.^5^ This complex crystallized readily following optimized crystallization conditions, as described previously and in the Materials and methods.^5^ Here, we used a spySrtA : peptide ratio equal to 1 : 2 (0.8 mM : 1 mM), which was increased as compared to our previous structures, at 1 : 1 (1 mM : 1 mM). Data was collected to 1.5 Å, and the structure was refined to a final *R*_work_/*R*_free_ = 19.3/21.5 (Fig. 5(A)). All data collection and refinement statistics are in Table 2.

**Table:** Crystal structure data collection and refinement statistics

	spySrtA-LPALA
Data collection	
Space group	*P*2_1_2_1_2_1_ (19)
Unit cell dimensions	
*a*, *b*, *c* (Å)	34.11, 57.48, 71.32
*α*, *β*, *γ* (°)	90, 90, 90
Resolution^a^ (Å)	35.7–1.5 (1.6–1.5)
*R*_sym_ b (%)	3.4 (35.5)
*I*/*σ*_I_^c^	27.20 (3.27)
Completeness (%)	99.3 (96.0)

Refinement	
Total # of reflections	23 034
Reflections in the test set	2299
*R*_work_ d/*R*_free_^e^	19.3/21.5
Number of atoms:	
Protein	1256
Water	126
Ramachandran plot^f^ (%)	100/0/0
*B*_av_ (Å^2^)	
Protein	17.1
Bond length RMSD (Å)	0.006
Bond angle RMSD (°)	0.916

PDB code	8T8G

a Values in parentheses are for data in the highest-resolution shell.

b
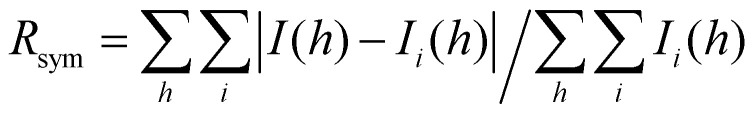
, where *I*_*i*_(*h*) and *I*(*h*) values are the *i*-th and mean measurements of the intensity of reflection *h*.

c SigAno = |*F*(+) − *F*(−)|/*σ*.

d *R*_work_ = Σ||*F*_obs_|_*h*_ − |*F*_calc_||_*h*_/Σ|*F*_obs_|_*h*_, *h* ∈ {working set}.

e *R*_free_ is calculated as *R*_work_ for the reflections *h* ∈ {test set}.

f Favored/allowed/outliers.

We saw clear electron density for the LPAL̲A peptide residues (Fig. 5(B)). Overall, the spySrtA-LPAL̲A structure agrees closely with our previously determined LPAT**A** and LPAT**S** structures (PDBs 7S51, 7S4O, respectively). Alignment revealed root-mean-square-deviation (RMSD) values for main chain atoms of 0.174–0.194 Å for the four protomers of the spySrtA-LPAT**A** and spySrtA-LPAT**S** structures, as compared to spySrtA-LPAL̲A (Fig. S4, ESI†). In addition, the peptides bind consistently in the peptide-binding cleft (Fig. S5A, ESI†). As predicted, the P1 Leu is stabilized by a hydrophobic pocket (the S1 site) previously described, and consisting of L113, L118, M125, A140, and V206 (Fig. 5(C)). These residues also interact with the Cγ atoms in the side chains of the P1 Thr in spySrtA-LPAT**A** and spySrtA-LPAT**S** (Fig. S5B, ESI†). This complex structure is also similar to other sortase-substrate structures solved (*e.g.*, PDB ID 2KID, 2RUI), with the caveat that the LPAT* peptidomimetic described does not include the P1 Thr carbonyl critical for catalysis, and is instead replaced with a sulfur atom that forms a disulfide bond not found in endogenous targets.^42,43^

Having confirmed our predictions about the stereochemistry of Leu binding in the S1 pocket, we were also curious if our structure would provide insight into why lmSrtA shows far lower levels of alternate cleavage. Our previous work revealed that the activity of lmSrtA for LPAL̲G and LPATG are equivalent (product conversion values of 85% and 88%, respectively).^22^ Consistently, alignment of our spySrtA-LPAL̲A structure, as well as frames from our spySrtA-LPATLA_shifted MD simulation, with lmSrtA (PDB 5U4)^27^ showed that lmSrtA has a hydrophobic pocket, comprised of residues L33 (not completely modeled in 5HU4), M40, A54, and I115 (crystal structure numbering), that can accommodate the P1 Leu (Fig. 5(D) and Fig. S6, ESI†). Notably, spySrtA L113, which was previously identified as making interactions with the P1 Leu is absent in lmSrtA; here, the equivalent residue is T28 (Fig. 5(D)). Also absent in lmSrtA, however, is an Ala in the β7–β8 loop to stabilize the puckered P1 Thr; lmSrtA contains T123 (5HU4 numbering) at this position in the loop, which is participating in an intraloop hydrogen-bond with D118 (5HU4 numbering) (Fig. 5(D)). Taken together, this suggests that while lmSrtA can accommodate a P1 Leu and perform standard cleavage of LPAL̲G, the alternative binding mode that facilitates alternative cleavage of LPATL is likely less favorable than in the case of spySrtA.

## Conclusions

*S. aureus* SrtA is the most well-studied sortase enzyme, and is highly selective for a CWSS with a P1′ Gly residue (LPAT**G**). However, work from ourselves and others suggests that this is not the case for other SrtA enzymes, specifically those from *Streptococcus* species.^22–25,44^ Recently, we made the observation that when the P1′ residue is a Leu, *S. pneumoniae* SrtA will preferentially cleave the peptide between the P1′/P2′ positions, in contrast to the canonical P1/P1′ site.^22^ Any type of non-standard cleavage or sequence recognition by sortase enzymes has the potential to be utilized in SML technologies; therefore, we were interested in understanding the stereochemical basis of this observation.

**Fig. 6:**
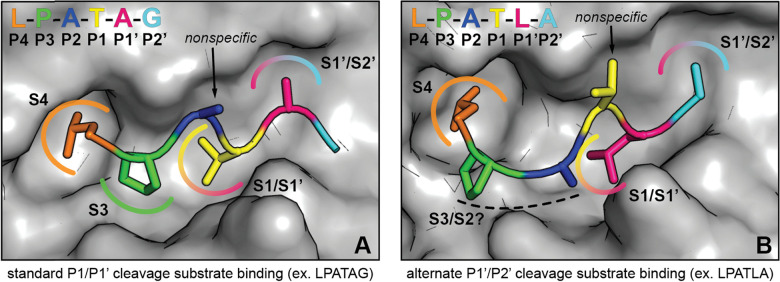
Comparison of substrate conformations leading to standard and alternate cleavage sites. (A) Illustration of spySrtA and bound substrate (LPATAG) in the standard cleavage conformation. The surface representation of C208A spySrtA (PDB 7S51) is shown in gray. The side chains of the bound substrate are shown as sticks, and the residues are colored as labeled. The P4 Leu and P3 Pro side chains are accommodated in hydrophobic pockets (S4 and S3, respectively), while the P3 side chain projects into solvent. For standard cleavage, the P1 Thr and P1′ Ala side chains occupy the same sites (S1/S1′ and S1′/S2′, respectively) that serve as binding sites for P1′ and P2′ in the alternate cleavage binding conformation. (B) Corresponding depiction of spySrtA and bound substrate (LPATLA) in the alternate cleavage conformation (spySrtA-LPATLA_shifted model). The protein surface and substrate are colored and labeled as in (A). The P4 Leu occupies the same pocket (S4) as in standard cleavage, while the P3 and P2 side chains occupy new positions that may confer varying levels of selectivity for different amino acids at these positions. The P1 Thr side projects into solvent, consistent with the relaxed substrate specificity at this site. Finally, the P1′ Leu and P2′ Ala side chains occupy the same positions as P1 and P1′ in the standard binding mode.

Here, we largely recapitulated our previous results using a related sortase enzyme, spySrtA. We also confirmed that lmSrtA has a greatly reduced ability to produce alternative cleavage products. We then used protein biochemistry, structural biology, and computational biochemistry to model and test an alternative binding mode whereby the P1′ Leu in the LPAT**L** motif binds in the S1 binding site of spySrtA (Fig. 6). As we predict that the P4 Leu interacts with spySrtA as it does under standard binding, this causes a pucker in the peptide, allowing for a unique orientation of P3, P2, and P1 residues (here, PAT). Our model of this binding mode for alternative cleavage does suggest that P2 selectivity is likely not as promiscuous as in the canonical LPXTG motif, however, as we see the P2 Ala in LPAT**L** does potentially makes hydrophobic contacts with spySrtA (Fig. 3(E)). This alternative binding mode also relaxes selectivity at the P1 position; therefore, substrates with amino acids (*e.g.* Glu) which result in no standard cleavage activity can participate in alternate cleavage if followed by Leu in P1′. Furthermore, we predict that stabilization of the P1 position in alternative binding relies on an interaction with the β7–β8 loop, which suggests why lmSrtA reacts poorly with LPAT**L**, despite having previously shown activity for LPAL̲G, with a P1 Leu.^22^

While the current study focuses on the reactivity of noncanonical substrates, it remains an open question as to how the inherent affinity of these substrates compares to standard LPXTG motifs. This was not explored in detail in this work, however our findings do suggest that the interactions of substrates with spySrtA, and likely other sortase enzymes, can be dominated by certain residues in the peptide sequence. We hypothesize that the P4 Leu and P3 Pro residues provide the bulk of the free energy of binding for these interactions. This conclusion is based on our data showing stable peptide binding at these positions even in simulations with sequences that are not efficiently cleaved by spySrtA, *e.g.*, LPAE̲G. This is also consistent with sortase-substrate structures from ourselves and others that show the canonical P1 Thr in different conformations (Thr-in and Thr-out), suggesting flexible binding in this position as opposed to the more rigid orientations of the P4 Leu and P3 Pro.^5,42,43^ Therefore, certain sequences, *e.g.*, LPAT**L**, may initially be recognized *via* binding of the LP amino acids, providing an opportunity for the P1′ Leu to dock in a favorable binding conformation in the S1 site. This alternative binding mode will result in P1′/P2′ cleavage, as that peptide bond will be properly positioned for catalysis. Under these conditions, the active site geometry is unaltered, but the stereochemistry of substrate binding is distinct.

The alternate cleavage mechanism described here represents a unique variation of the otherwise standard transpeptidation pathway catalyzed by class A sortases with LPXTG substrates. This mode of reactivity potentially has uses in the context of SML technology, where recent work has highlighted the utility of sortase-substrate pairs that offer alternatives to the canonical LPXTG motif.^8,45,46^ This work also contributes to the fundamental understanding of substrate recognition by sortases, providing further evidence that sortases within the same class can exhibit significant differences in substrate selectivity. We anticipate that a thorough understanding of these differences, particularly at the structural level, may prove valuable for both the continued development of SML and the design of therapeutics that target sortases *in vivo*.

## Author contributions

Conceptualization: JMA, JFA; investigation: BAV, JMB, HMK, NJGR, DJA, SNJ; data curation: BAV, JMB, HMK, DA, SNJ, JFA; writing – original draft: JFA, writing – reviewing & editing: BAV, JMB, HMK, NJGR, DJA, SNJ, JMA, JFA; funding acquisition: JMB, JMA, JFA.

## Conflicts of interest

The authors declare no conflicts of interest.

## Supplementary Material

CB-005-D3CB00129F-s001
