# Supplementary material for: A unique binding mode of P1′ Leu-containing target sequences for Streptococcus pyogenes sortase A results in alternative cleavage
Source: RSC Chem Biol. 2023 Oct 18;5(1):30–40. doi: 10.1039/d3cb00129f (PMC10763551; doi:10.1039/d3cb00129f)
Supplement: CB-005-D3CB00129F-s001 [file CB-005-D3CB00129F-s001.pdf]

Electronic Supplementary Material (ESI) for RSC Chemical Biology.
This journal is © The Royal Society of Chemistry 2023

Supplementary Information for:

A unique binding mode of P1' Leu-containing target sequences for *Streptococcus pyogenes* Sortase A results in alternative cleavage

Brandon A. Vogel,[1,†] Jadon M. Blount,[1,†] Hanna M. Kodama,[1,†] Noah J. Goodwin-Rice,[1] Devin J. Andaluz,[1] Sophie N. Jackson,[1] John M. Antos,[1,*] Jeanine F. Amacher[1,*]

[1]Department of Chemistry, Western Washington University, Bellingham, WA, 98225
[†]These authors contributed equally to this work.

*Corresponding Authors:

John Antos
Email: antosj@wwu.edu

Jeanine Amacher
Email: amachej@wwu.edu

This PDF file includes:

Tables S1 to S2 (pp 2-3)
Figures S1 to S6 (pp 4-9)

Table of Contents

Table S1. LC-ESI-MS characterization of proteins, intact peptide substrates, and Dnp-containing reaction products. 2

Table S2. Molecular dynamics parameters for all simulations. 3

Figure S1. Post-equilibration starting models for MD simulations. 4

Figure S2. MD simulation main chain fluctuations over 1000 ns. 5

Figure S3. Analysis of transacylation products derived from LPAEL and LPATL substrates. 6

Figure S4. Structural alignments of spySrtA complex structures. 7

Figure S5. Structural comparison of all spySrtA-peptide bound structures. 8

Figure S6. Structural alignment of spySrtA-LPALA and lmSrtA. 9

Table S1. LC-ESI-MS characterization of proteins, intact peptide substrates, and Dnp-containing reaction products.

Protein[a]	Expected Mass (Da)	Observed Mass (Da)
WT spySrtA	20657.4	20657.4
H143A spySrtA	20591.3	20591.2
I211P spySrtA	20641.3	20642.1
$spySrtA_{monocytogenes}$	20798.6	20798.6
WT lmSrtA	18070.5	18070.5
$lmSrtA_{pyogenes}$	18810.2	18810.3

Substrates/Products[b]	Expected Mass (m/z)	Observed Mass (m/z)
**Abz-LPATGGK(Dnp)**	927.4	927.4
GGK(Dnp), standard cleavage product	426.2	426.2
GK(Dnp), alternative cleavage product	369.2	not observed
**Abz-LPATLGK(Dnp)**	983.5	983.5
LGK(Dnp)	482.2	482.2
GK(Dnp)	369.2	369.2
**Abz-LPATYGK(Dnp)**	1033.5	1033.5
YGK(Dnp)	532.2	532.2
GK(Dnp)	369.2	369.2
**Abz-LPAEGGK(Dnp)**	955.4	955.4
GGK(Dnp)	426.2	426.2
GK(Dnp)	369.2	369.2[c]
**Abz-LPAELGK(Dnp)**	1011.5	1011.5
LGK(Dnp)	482.2	not observed[d]
GK(Dnp)	369.2	369.2

[a]Expected and observed masses for proteins represent average molecular weight (Da). Observed mass values for proteins were determined from deconvolution of protein charge ladders observed in each ESI-MS spectrum. [b]Expected and observed values for peptide substrates and reaction products represent $[M+H]^+$ ions (monoisotopic). [c]Only trace levels observed by LC-MS in reactions between Abz-LPAEGGK(Dnp) and WT spySrtA. [d]This product was not observed in model reactions employing excess hydroxylamine as the reaction nucleophile. Trace levels were observed in reactions utilizing glycinamide as the reaction nucleophile (see **Fig. S3C**).

Table S2. Molecular dynamics parameters for all simulations.

System	Number of Atoms	Cubic box dimensions (nm)	Simulation time (ns)
spySrtA-LPATLA_shifted	34121	7.019	1000
spySrtA-LPALA	34125	7.019	1000
spySrtA-LPAEG	34128	7.019	1000
spySrtA-LPAELA	40773	7.019	1000

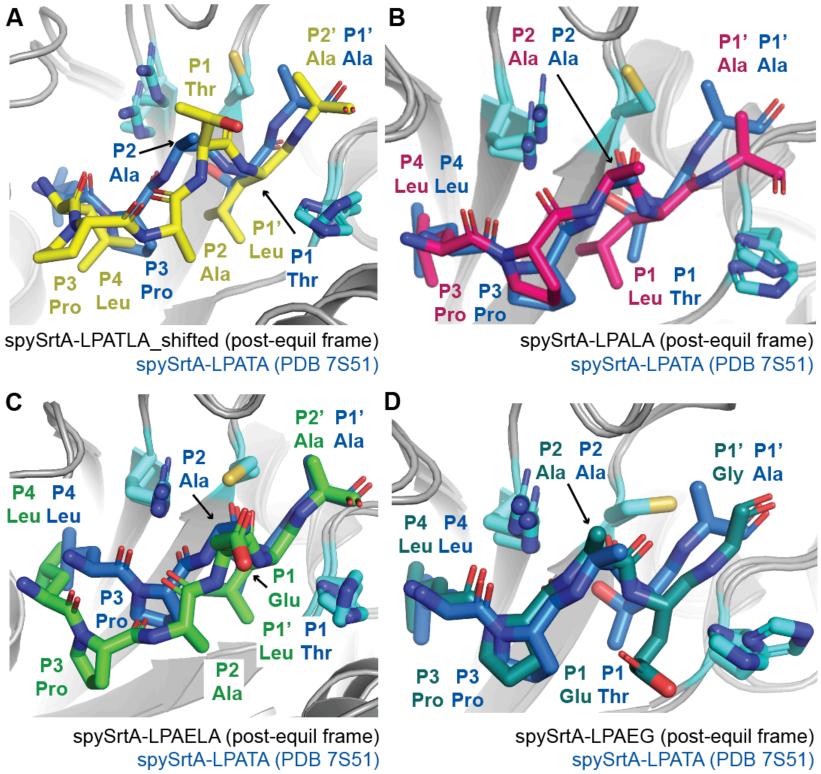

Figure S1. Post-equilibration starting models for MD simulations. (A-D) The starting models, post-equilibration, are shown for each of the four MD simulations, spySrtA with: LPATLA_shifted (A), LPALA (B), LPAELA (C), and LPAEG (D). In all, spySrtA is in gray cartoon, with the catalytic residues shown as side chain sticks and colored by heteroatom (C=cyan, N=blue, S=yellow). The spySrtA-LPATA structure (PDB 7S51) is used to compare (blue sticks, colored by heteroatom), and RMSD values for each main chain alignment are: 0.717 Å over 555 atoms (A), 0.677 Å over 550 atoms (B), 0.837 Å over 563 atoms (C), and 0.866 Å over 571 atoms (D). Peptides are colored as labeled for each, with carbons as: LPATLA_shifted = yellow (A), LPALA = pink (B), LPAELA = green (C), and LPAEG = teal (D).

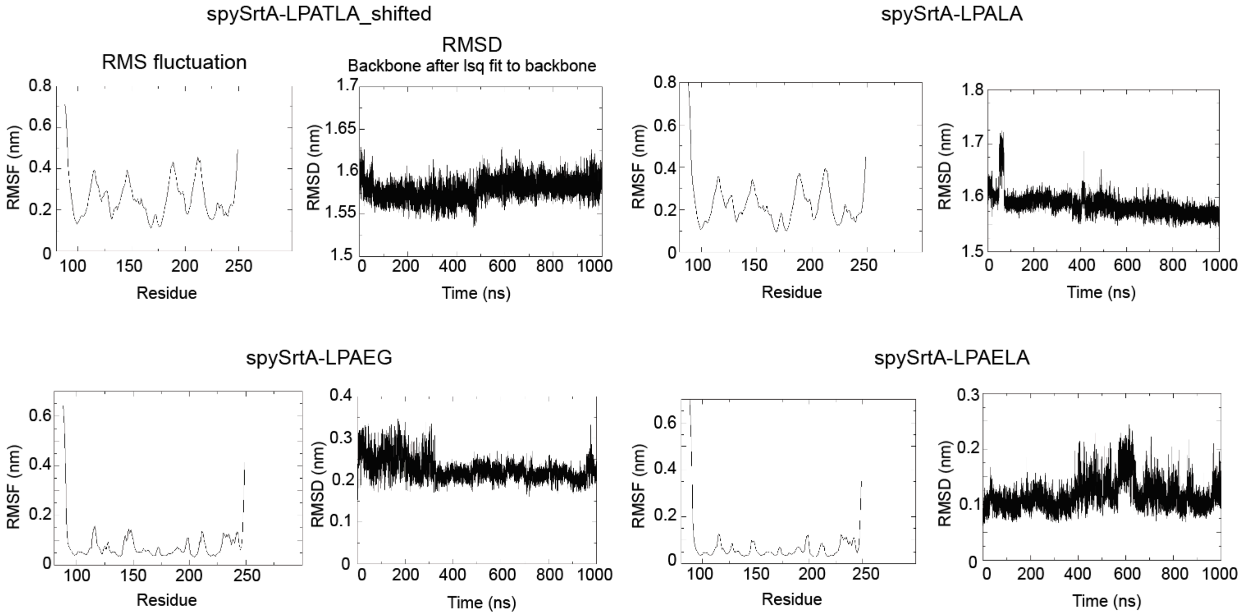

Figure S2. MD simulation main chain fluctuations over 1000 ns. The average root-mean-square-fluctuation (RMSF) from the average position for the main-chain sortase atoms over the course of each simulation is graphed for each simulation, as labeled (left-hand graphs). RMSF values are as expected, based on known secondary structure elements and flexible loop regions. The average root-mean-square-deviation (RMSD) for sortase backbone atoms over the course of the simulation is graphed for all four simulations, as labeled.

A

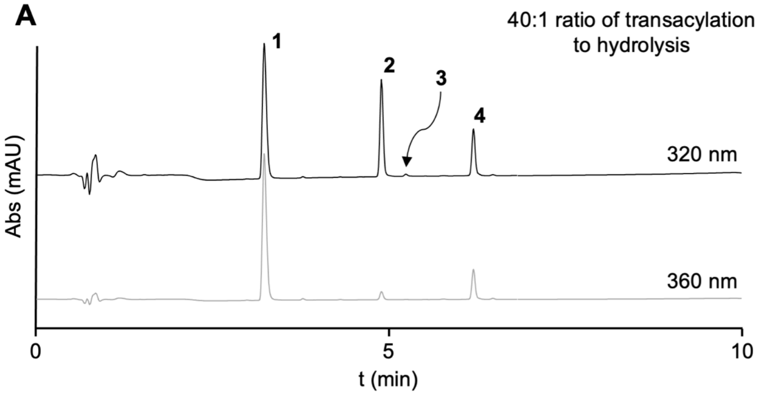

Peak	Identity	Exp (m/z)	Obs (m/z)
1	GK(Dnp)	369.2	369.2
2	Abz-LPAEL-*NHOH*	676.4	676.4
3	Abz-LPAEL-*OH*	661.4	661.4
4	Abz-LPAELGK(Dnp)	1011.5	1011.5

B

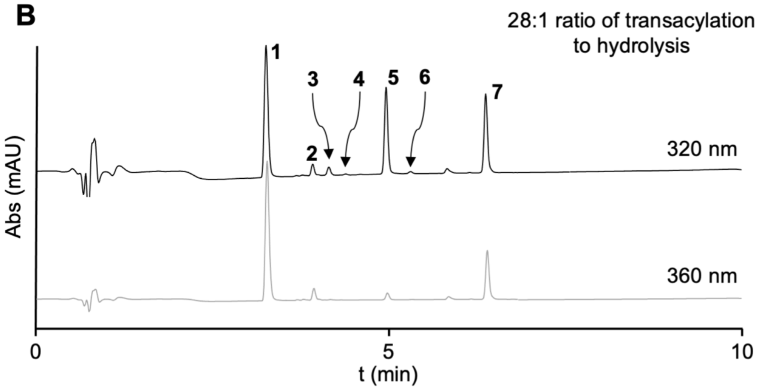

Peak	Identity	Exp (m/z)	Obs (m/z)
1	GK(Dnp)	369.2	369.2
2	LGK(Dnp)	482.2	482.2
3	Abz-LPAT-*NHOH*	535.3	535.3
4	Abz-LPAT-*OH*	520.3	520.3
5	Abz-LPATL-*NHOH*	648.4	648.4
6	Abz-LPATL-*OH*	633.4	633.4
7	Abz-LPATLGK(Dnp)	983.5	983.5

C

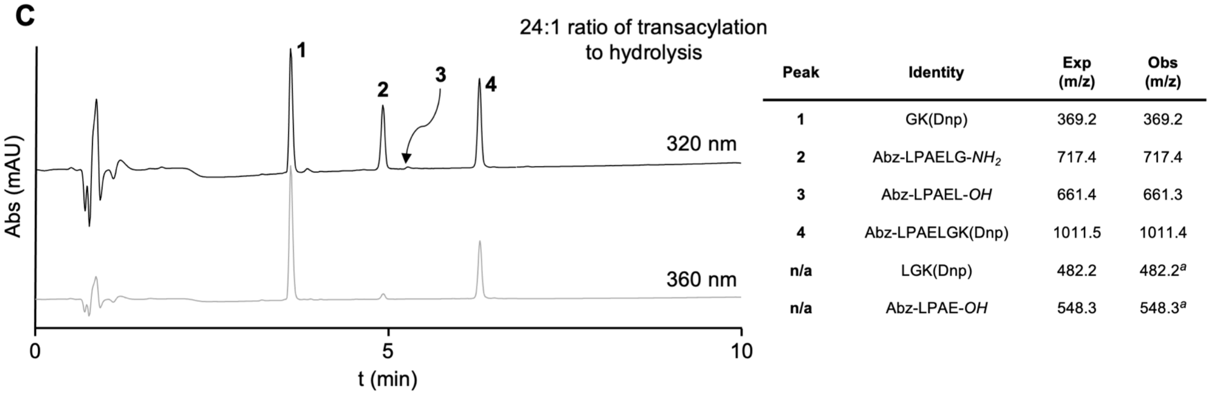

Peak	Identity	Exp (m/z)	Obs (m/z)
1	GK(Dnp)	369.2	369.2
2	Abz-LPAELG-NH_2	717.4	717.4
3	Abz-LPAEL-*OH*	661.4	661.3
4	Abz-LPAELGK(Dnp)	1011.5	1011.4
n/a	LGK(Dnp)	482.2	482.2[a]
n/a	Abz-LPAE-*OH*	548.3	548.3[a]

Figure S3. Analysis of transacylation products derived from LPAEL and LPATL substrates. Representative RP-HPLC (320/360 nm) chromatograms for the reactions of (A) wild-type spySrtA with Abz-LPAELGK(Dnp) and excess hydroxylamine at 18 h, (B) wild-type spySrtA with Abz-LPATLGK(Dnp) and excess hydroxylamine at 18 h, and (C) wild-type spySrtA with Abz-LPAELGK(Dnp) and excess glycinamide at 24 h. Chromatograms at 320 nm highlight the presence of transacylation/hydrolysis products that only contain the Abz moiety, which otherwise do not absorb strongly at 360 nm. Peak tables are included with each pair of chromatograms, including associated LC-MS data used to confirm the identity of each peak. Expected (exp) and observed (obs) m/z values represent $[M+H]^+$ ions (monoisotopic). Transacylation products for panels A and B contain C-terminal hydroxamides (indicated as -*NHOH*). The transacylation product for panel C contains a C-terminal primary amide (-NH_2) arising from the glycinamide nucleophile. Hydrolysis products are indicated with -*OH* to signify the C-terminal carboxylic acid. [a]Trace levels of species arising from cleavage at the P1/P1' site were observed by LC-MS, but were otherwise undetectable in the UV chromatograms.

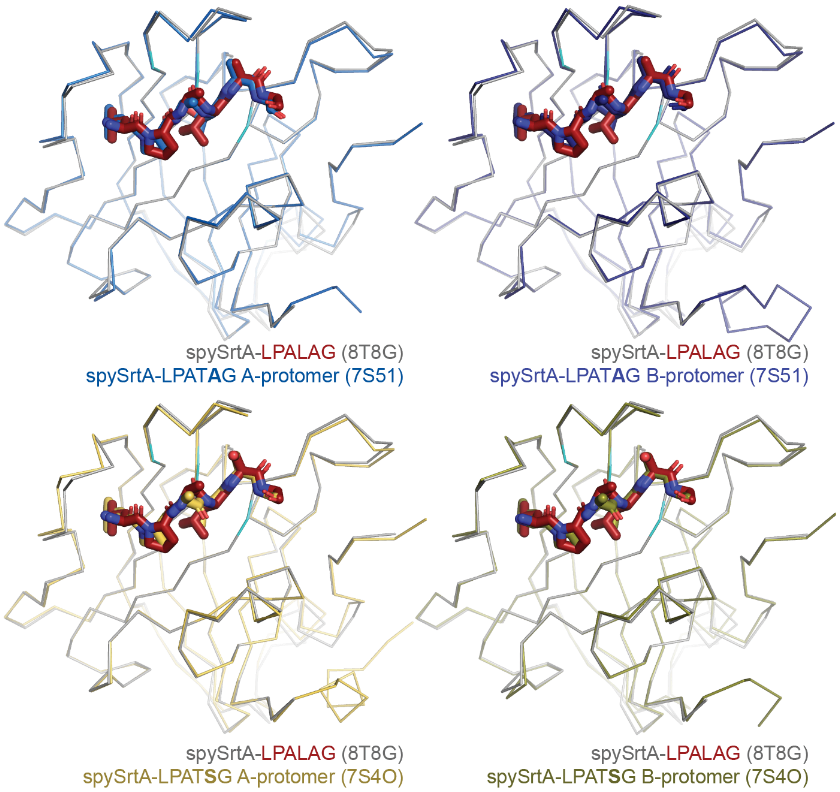

Figure S4. Structural alignments of spySrtA complex structures. For all, the spySrtA protein is shown in ribbon/Cα trace representation. The LPALAG peptide is in dark red sticks and colored by heteroatom (N=blue, O=red). The A- and B-protomers of the spySrtA-LPATAG (PDB 7S51, below as 7S51-A and 7S51-B) and spySrtA-LPATSG (PDB 7S4O, below as 7S4O-A and 7S4O-B) are colored as labeled, with peptides as sticks. Alignment RMSD values for main chain atoms are: 7S51-A = 0.194 Å over 484 atoms, 7S51-B = 0.180 Å over 470 atoms, 7S4O-A = 0.174 Å over 472 atoms, and 7S4O-B = 0.192 Å over 490 atoms.

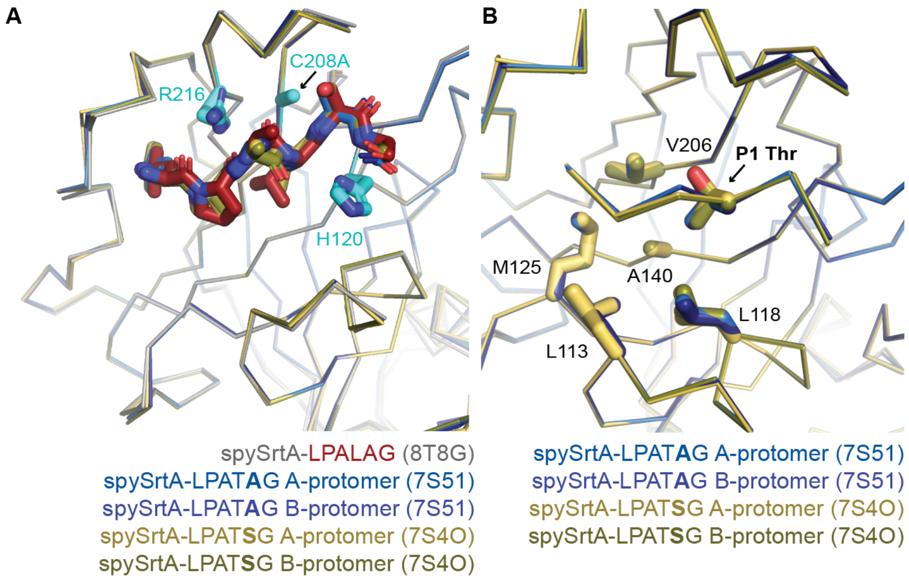

Figure S5. Structural comparison of all spySrtA-peptide bound structures. (A) Structural alignment of all spySrtA-peptide bound protomers (PDBs 7S51, 7S4O, and 8T8G from this work). The spySrtA backbone is in ribbon/Cα trace representation, and colored as labeled. Peptides are shown as sticks and colored by heteroatom (C=as labeled, N=blue, O=red). The catalytic triad side chain atoms are in cyan sticks and colored by heteroatom. (B) A conserved hydrophobic binding pocket for the P1 position (here, Thr) is shown for the spySrtA-LPATA (PDB 7S51) and spySrtA-LPATS (PDB 7S4O) structures. Rendering is as in (A), which relevant side chain sticks shown and labeled.

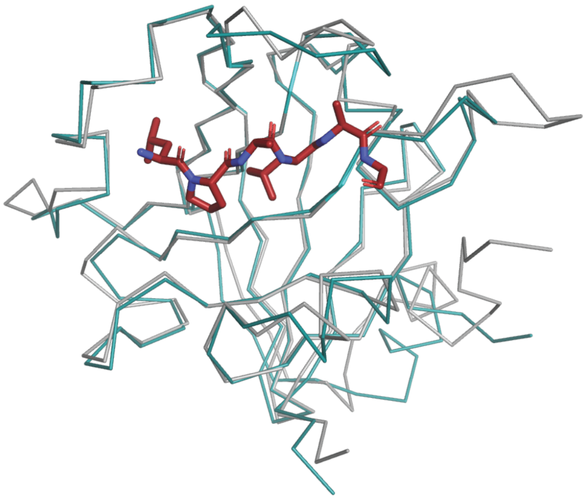

Figure S6. Structural alignment of spySrtA-LPALA and lmSrtA. The spySrtA (gray) and lmSrtA (teal, PDB 5HU4) proteins are shown in ribbon/Cα trace representation. The LPALAG peptide is in dark red sticks, colored by heteroatom (N=blue, O=red). The spySrtA and lmSrtA main chain atoms aligned with an overall RMSD of 0.494 Å over 397 atoms.
